# Synergistic Roles of Curcumin in Sensitising the Cisplatin Effect on a Cancer Stem Cell-Like Population Derived from Non-Small Cell Lung Cancer Cell Lines

**DOI:** 10.3390/molecules26041056

**Published:** 2021-02-18

**Authors:** Nazilah Abdul Satar, Mohd Nazri Ismail, Badrul Hisham Yahaya

**Affiliations:** 1 Lung Stem Cell and Gene Therapy Group, Regenerative Medicine Cluster, Advanced Medical and Dental Institute (IPPT), Universiti Sains Malaysia, Sains@Bertam Kepala Batas, Penang 13200, Malaysia; nazilah_satar@yahoo.com; 2 Analytical Biochemistry Research Centre (ABrC), Universiti Sains Malaysia, Penang 11800, Malaysia; mdnazri@usm.my

**Keywords:** lung cancer stem cells, curcumin, cisplatin, sensitisation, preventive, non-small cell lung cancer

## Abstract

Cancer stem cells (CSCs) represent a small subpopulation within a tumour. These cells possess stem cell-like properties but also initiate resistance to cytotoxic agents, which contributes to cancer relapse. Natural compounds such as curcumin that contain high amounts of polyphenols can have a chemosensitivity effect that sensitises CSCs to cytotoxic agents such as cisplatin. This study was designed to investigate the efficacy of curcumin as a chemo-sensitiser in CSCs subpopulation of non-small cell lung cancer (NSCLC) using the lung cancer adenocarcinoma human alveolar basal epithelial cells A549 and H2170. The ability of curcumin to sensitise lung CSCs to cisplatin was determined by evaluating stemness characteristics, including proliferation activity, colony formation, and spheroid formation of cells treated with curcumin alone, cisplatin alone, or the combination of both at 24, 48, and 72 h. The mRNA level of genes involved in stemness was analysed using quantitative real-time polymerase chain reaction. Liquid chromatography-mass spectrometry was used to evaluate the effect of curcumin on the CSC niche. A combined treatment of A549 subpopulations with curcumin reduced cellular proliferation activity at all time points. Curcumin significantly (*p* < 0.001) suppressed colonies formation by 50% and shrank the spheroids in CSC subpopulations, indicating inhibition of their self-renewal capability. This effect also was manifested by the down-regulation of *SOX2*, *NANOG*, and *KLF4*. Curcumin also regulated the niche of CSCs by inhibiting chemoresistance proteins, aldehyde dehydrogenase, metastasis, angiogenesis, and proliferation of cancer-related proteins. These results show the potential of using curcumin as a therapeutic approach for targeting CSC subpopulations in non-small cell lung cancer.

## 1. Introduction

Non-small cell lung cancer (NSCLC) is the most common type of lung cancer as it accounts for approximately 80–85% of all lung cancer cases, with the remainder being small cell lung cancer (SCLC) [1,2,3,4]. Conventional treatments such as radiotherapy, chemotherapy, and surgery are common cancer therapy methods, but they often lead to tumour recurrence. This recurrence is mainly because conventional therapies only target the bulk of the tumour, leaving cancer stem cells (CSCs) behind, and they are believed to be the driving force of tumour progression [5,6,7,8]. CSCs are a subset of a tumour [2,9] as they mimic stem cell features, including active proliferation and the ability to metastasise, self-renew, and differentiate. Self-renewal is a crucial component of CSC, as it maintains the stemness and its pool. CSCs also sheltered within the cancerous microenvironment, called the niche. This niche helps them retain their self-renew ability and propagate more differentiated progenitor cells while staying undifferentiated state [10,11]. Therefore, eliminating CSCs could improve therapeutic strategies, especially in NSCLC. Recent studies have suggested that natural polyphenols might be used to sensitise tumour cells to chemotherapy and radiotherapy by inhibiting the pathway that leads to treatment resistance. Curcumin (diferuloylmethane) is a naturally occurring polyphenol extract that is found in turmeric. Curcumin has long been used as a food, cosmetic, and traditional herbal medicine. Significant evidence indicated that curcumin’s anti-cancer potential against many types of cancer, including breast, pancreas, prostate, lung, melanoma, and head and neck cancers [12,13]. Unlike many ‘targeted’ chemotherapeutic drugs that suffer from toxicity and resistance, curcumin by itself can target specific molecules and pathways without any associated toxicity or resistance [14]. One of the most compelling reasons for exploring curcumin is its sensitiser properties, which influence a diverse range of molecular targets within cells. Combining curcumin with chemotherapy drugs led to the hypothesis that efficacy could be enhanced by adding two or more targeted agents to combat cancer cells’ resistance mechanism. Our previous study has shown that curcumin was able to increase the efficacy of cisplatin by enhancing the cisplatin-induced metastatic inhibition and apoptosis of the highly migratory CSC subpopulation of NSCLC cell lines. However, there was still an unknown mechanism on how curcumin regulates cisplatin that had resulted in sensitising the CSCs microenvironment that later inhibits the tumour progression and reducing metastasis [15]. Our previous study has also shown that CSCs exhibit the characteristics of multipotent stem cells and their genetic composition similar to that of normal stem cells, thus could be potential therapeutic target to treat lung cancer [16,17]. Therefore, the goal of this study was to determine the mechanism of how curcumin sensitises CSCs to cisplatin by evaluating the sensitisation effect of curcumin on various aspects of self-renewal and proliferation assays. The identification of proteins that regulate the CSC niche of NSCLC would open up future targeted therapy for lung cancer.

## 2. Results

### 2.1. Curcumin Sensitises the Cisplatin Effect Leading to Decreased Cells in

#### 2.1.1. Proliferation Activity 

**Figure 1:**
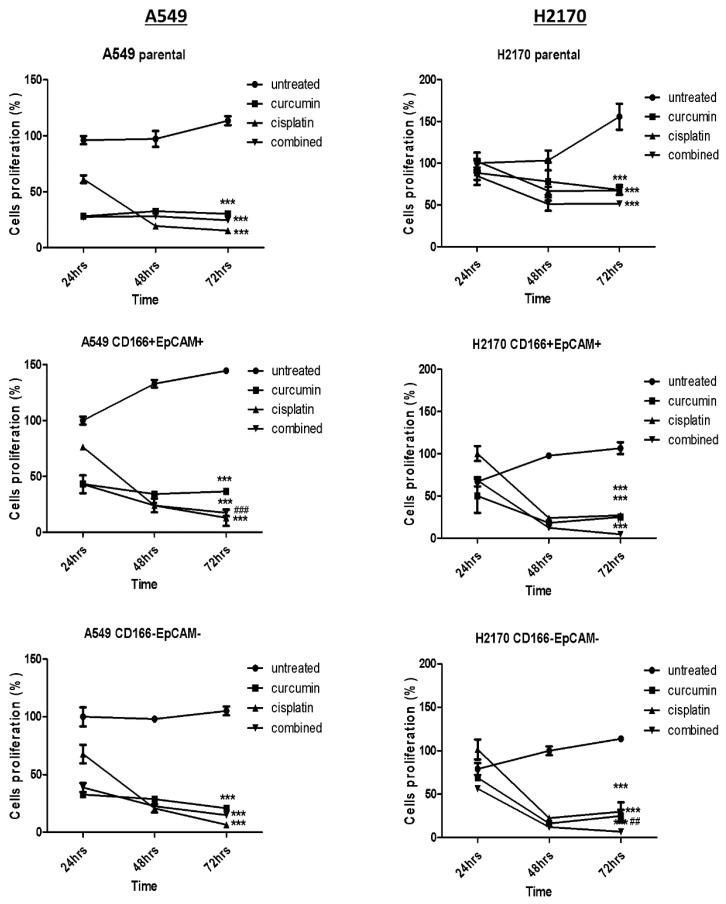
Cell proliferation activity of NSCLC CSCs of A549 and H2170 cells before single and combination treatment after 24 h, 48 h, and 72 h. Each bar represents the average mean ± SD of triplicate samples. Statistical significance was measured with the two-way ANOVA. *** *p* < 0.001 compared with untreated cells. ## *p* < 0.01, ### *p* < 0.001 compared with curcumin alone.

As reported in our previous study [15], the combination of 41 μM curcumin and 30 μM cisplatin was selected for A549 cells, and 33 μM of curcumin and 7 μM of cisplatin were selected for H2170 cells for further downstream study. The effect of curcumin in sensitising cisplatin’s effect was further evaluated on the proliferation activity of the CSCs by using single or in the combination of treatment on CSCs of A549 and H2170 cell lines at 24 h, 48 h, and 72 h post-treatment. Due to the heterogeneity of parental cells, both A549 and H2170 parental cells were used as a control. As shown in Figure 1, a single treatment of curcumin and cisplatin was significantly (*p* < 0.001) reduced proliferation activity in both A549 CD166 + EpCAM + and H2170 CD166 + EpCAM + CSCs subpopulations following 24 h, 48 h, and 72 h post-treatment, respectively. Furthermore, the cell proliferation activities were further reduced in a combined treatment (*p* < 0.05). The effect of the treatment on cancer cells’ proliferation activity was also seen in the non-CSCs population of both cancer cell lines.

#### 2.1.2. Cell Cycle Regulation in CSCs of NSCLC

To examine the mechanism by which curcumin regulated cell proliferation, cell cycle analysis was conducted to observe the distribution of cell cycle events in CD166 + EpCAM + CSCs subpopulation after treatment with the single and combined doses of curcumin and cisplatin. Both synergistic (rescue) and sensitisation (preventive) effects were conducted on both CD166 + EpCAM + CSCs subpopulation and CD166-EpCAM- non-CSCs subpopulations.

##### Curcumin Arrests the Cell Cycle in CSCs Subpopulation by Synergistic and Sensitisation Treatment Groups

**Figure 2:**
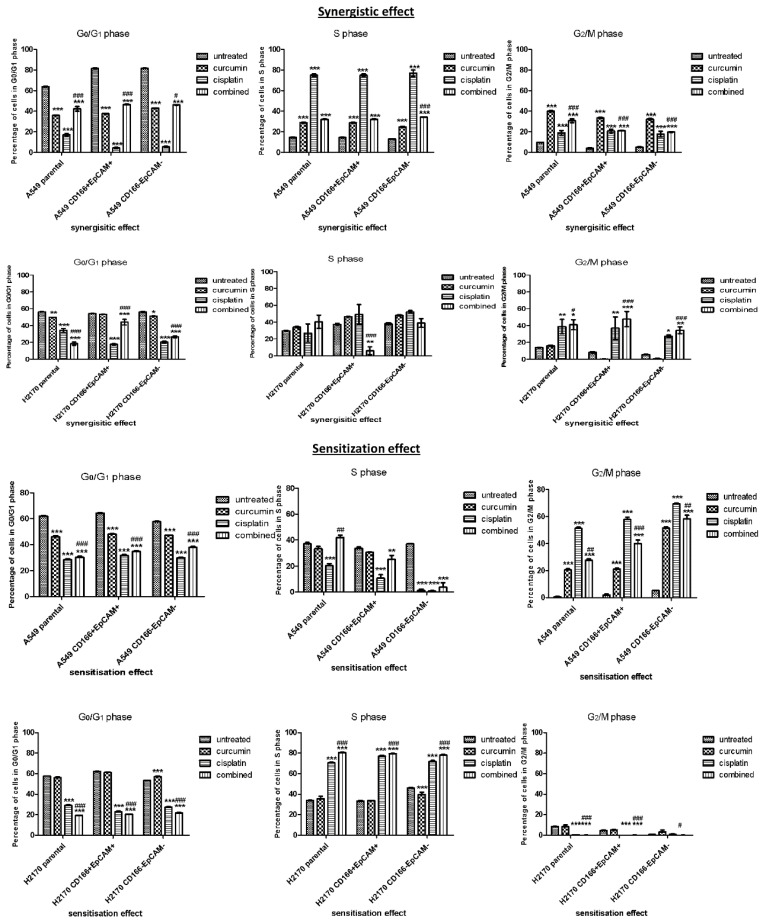
Effect of curcumin on cell cycle distribution on CD166 + EpCAM + CSCs subpopulation and CD166-EpCAM- non-CSCs subpopulation of A549 and H2170 cell lines in both rescue (synergistic) and preventive (sensitisation) treatment groups. In rescue (synergistic) treatment group, the cells were treated with single and combined treatment on day 7 after the formation of colonies or spheroids. In the preventive (sensitisation) treatment groups the cells were treated with single and combined treatment, 48 h post-treatment. Cell cycle analysis was performed by flow cytometry. Each bar represents the average mean ± SD of triplicate samples. Statistical significance was measured with the two-way ANOVA. * *p* < 0.05, ** *p* < 0.01, *** *p* < 0.001 compared with untreated cells. # *p* < 0.05, ## *p* < 0.01, ### *p* < 0.001 compared with curcumin alone.

In the synergistic effect, the A549 CD166 + EpCAM + and H2170 CD166 + EpCAM + CSCs subpopulations were treated with curcumin and cisplatin simultaneously for 48 h. Treated cells were harvested and processed for flow cytometry analysis. The cell cycle analysis (Figure 2) revealed that in the A549 population, either single or combined treatment, the addition of curcumin in the treatment had significantly inhibited (*p* < 0.001) the G0/G1 phase when compared to cisplatin alone. A similar pattern is also shown in H2170 cell populations in the G0/G1 phase of the cell cycle. However, treatment with cisplatin demonstrated a significantly arrested in S-phase (*p* < 0.001). In H2170 cells, the CD166 + EpCAM + CSCs subpopulations showed a significant S-phase was arrested in a single treatment of both curcumin and cisplatin (*p* < 0.001). At the G2/M phase, either single or combined treatment with cisplatin, the presence of curcumin was significantly arrested the G2/M phase, even when compared to untreated cells (*p* < 0.001). There was no significant effect of single treatment with curcumin at the G2/M phase in H2170 cell populations; however, when cisplatin was synergistically combined with curcumin, the percentage of G2/M phase was elevated.

In the sensitisation treatment group, in the case of combined treatment, the cells were initially exposed to curcumin before treated with cisplatin. This initiative has demonstrated the increasing percentage of cell cycle arrest at both G0/G1 and S phases in A549 cell populations. However, in the H2170 cell population, the significant effect of combined treatment was slightly higher than single treatment with cisplatin and curcumin alone. In the A549 cell population, treatment with cisplatin alone and combined with curcumin demonstrated a significant effect compared to curcumin alone (*p* < 0.05)—Figure 2.

### 2.2. Curcumin Suppresses and Prevents the Self-Renewal Characteristics of CSCs Subpopulation

To study the effect of curcumin on self-renewal characteristics of the CD166 + EpCAM + CSCs subpopulation, the colonies and sphere-forming assays were performed to evaluate the ability of curcumin in suppressing the self-renewal capability in CSCs subpopulation. Besides, the study has also investigated the potential of curcumin to suppress (rescue treatment) the self-renewal and prevent (prevention treatment) the self-renewal capability in CSCs subpopulation in both colonies development and spheroid formation.

#### 2.2.1. Curcumin Suppresses and Prevents the Colonies Formation

**Figure 3:**
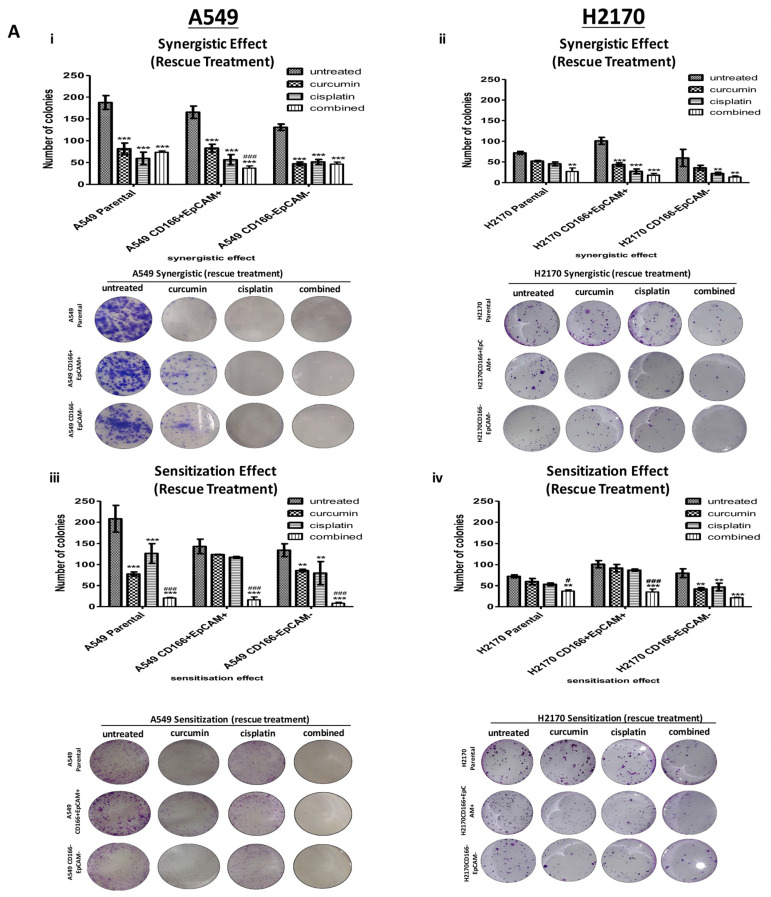

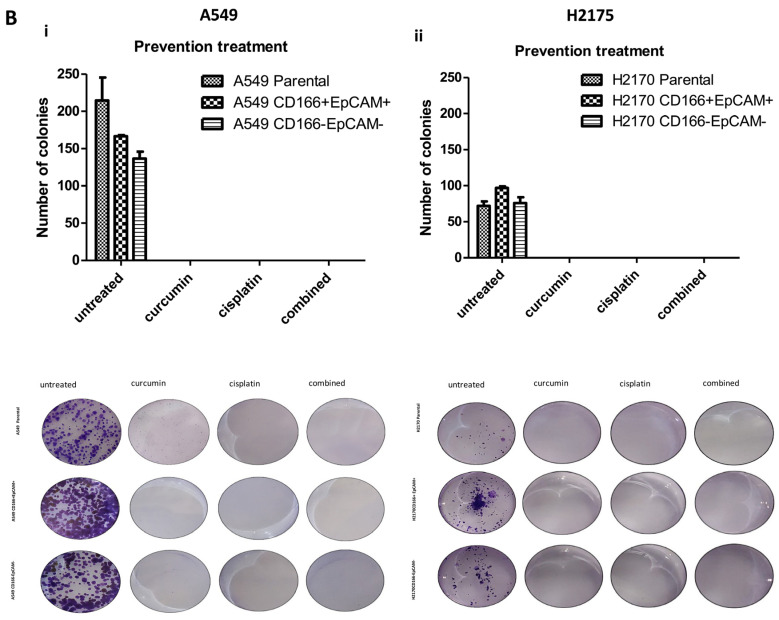
The effect of curcumin on colonies formation as the rescue and the preventive agents. (**A**): Curcumin suppresses colonies formation using both synergistic (**i**,**ii**) and sensitisation (**iii**,**iv**) assays in both CD166 + EpCAM + CSCs subpopulation and CD166EpCAM- non-CSCS subpopulations of A549 and H2170 cells. In this experiment, the colonies were allowed to develop before the treatment with curcumin was given. (**B**): Curcumin acts as preventive agent where the cells were treated with curcumin before colonies were developed. Each bar represents the average mean ± SD of triplicate samples. Statistical significance was measured with the two-way ANOVA. ** *p* < 0.01, *** *p* < 0.001 compared with untreated cells. # *p* < 0.05, ### *p* < 0.001 compared with curcumin alone.

The A549 and H2170 parental cells were used as a control to indicate the basal level of self-renewal inhibition in a heterogeneous population. In this rescue treatment, the colonies were allowed to grow for 7 and 14 days, and once colonies were formed, the treatments were given for 48 h before the experiment end. Subsequently, the formation of colonies was evaluated using crystal violet staining to determine the colonies’ number. In synergistic effect Figure 3A(i,ii), the single treatment of curcumin was found to inhibit the colonies formation at 50–57% both in A549 CD166 + EpCAM + and H2170 CD166 + EpCAM + CSCs subpopulations, thus suggesting that curcumin alone can inhibit the self-renewal capability in CSCs population. The combined treatment markedly enhanced the colonies’ inhibition by up to 78% (37 colonies) and 82% (18 colonies) of the colonies formation in all A549 and H2170 cell populations.

In the sensitisation effect, the single treatment of curcumin and cisplatin has only resulted in 25% (124 colonies) and 29% (117 colonies) of colonies inhibition in the A549 CD166 + EpCAM + CSCs subpopulation. However, when the A549 CD166 + EpCAM + CSCs subpopulation was exposed to a high dose of curcumin followed by a low dose of cisplatin in combined treatment, a total of colonies inhibition increased to 90% (20 colonies) as depicted in Figure 3A(iii,iv), which was suggested that curcumin sensitisation was very effective in suppressing the self-renewal capability of CSCs. A similar pattern was also observed in the H2170 CD166 + EpCAM + CSCs subpopulation in a combined treatment. Interestingly, colony formation inhibition is not limited to the CSCs population but also observed in the non-CSC population.

In the preventive treatment group, curcumin as a preventive agent was investigated by treating the cells with curcumin before developing colonies or spheroid, which was speculated to be effective in preventing the self-renewal capability. Interestingly, as shown in Figure 3B(i,ii), no colonies were observed in all treatment groups of both A549 and H2170 cell populations.

#### 2.2.2. Curcumin Suppresses and Prevents Spheroid Development

To get more conclusive data on the effect of curcumin on CSCs, a more sensitive non-adherent 3D sphere assay was used to confirm that curcumin’s action inhibited the self-renewal activity. Similarly, in this rescue treatment, the CD166 + EpCAM + CSCs and CD166-EpCAM- non-CSCs subpopulations of both A549 and H2170 cells were grown anchorage-independent spheres at a certain period, then the cells were treated either with single (curcumin and cisplatin) or combined treatment until day-21.

**Figure 4:**
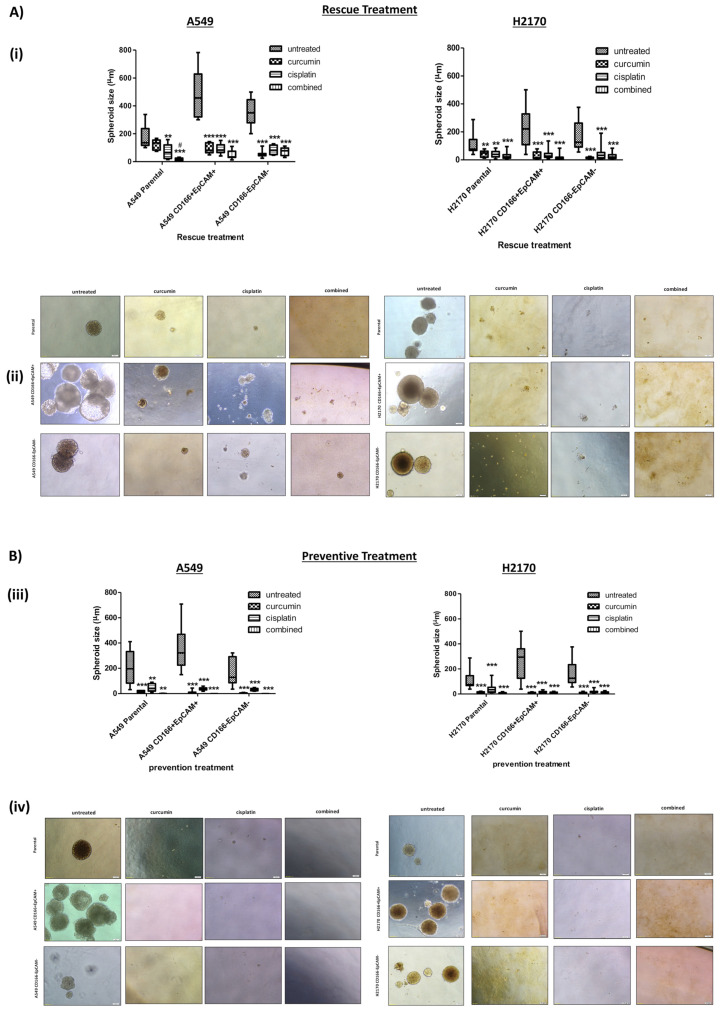
(**A**,**B**): Curcumin inhibits the spheroid formation of the CD166 + EpCAM + CSCs subpopulation and A549 CD166-EpCAM- non-CSCs subpopulation of A549 and H2170 cells in rescue (**A**) and preventive (**B**) treatment groups. (**i**) Boxplot illustrated the sphere size analysis. Upper and lower boxplot margins represent the interquartile range, and the middle bar indicated the median. The whisker defines the range of values. (**ii**) Representative photomicrographs of spheroid formation are presented for both CD166 + EpCAM + CSCs subpopulation and A549 CD166-EpCAM- non-CSCs subpopulation of A549 and H2170 cells at ×20 magnification. In the rescue treatment group, spheroid cells were treated with single and combined treatment and were evaluated after day-21. While in the preventive treatment group, cells were treated with either single or combined treatment after 72 h of cultured and were continued growing the colonies until 14 days before staining. Each bar represents the average mean ±SD of triplicate samples. Statistical significance was measured with the two-way ANOVA. ** *p* < 0.01, *** *p* < 0.001 compared with untreated cells. # *p* < 0.05 compared with curcumin alone.

As depicted in Figure 4A, the A549 CD166 + EpCAM + and H2170 CD166 + EpCAM + CSCs subpopulations had developed a larger spheroid with an average diameter of 502.6 µm and 236.1 µm, respectively as compared to A549 CD166-EpCAM- and H2170 CD166-EpCAM- non-CSCs subpopulations with 352.1 µm and 178.1 µm respectively. The result demonstrated that incubating the spheroid with a single treatment of curcumin had led to shrinkage of spheroid diameter to an average of 91.76 µm in the A549 CD166 + EpCAM + CSCs subpopulation and 30.9 µm in the H2170 CD166 + EpCAM + CSCs subpopulation. As expected, the combined treatment had caused a significant (*p* < 0.001) inhibition of spheroid diameter by 48.3 µm and 17.8 µm in both A549 CD166 + EpCAM + and H2170 CD166 + EpCAM + CSCs subpopulations. A549 CD166-EpCAM- and H2170 CD166-EpCAM- non-CSCs subpopulations also showed a massive inhibitory effect since non-CSCs is more sensitive less resistant as compared to CSCs subpopulation.

In the preventive effect, cells were treated for 48 h either through synergistic effect before subjected to colonies or spheroid assay to evaluate for self-renewal capability. Interestingly, as shown in Figure 4B, no spheroid formation was observed in all treatment groups for both A549 and H2170 cell lines.

### 2.3. Curcumin Regulated Stemness-Related Genes in CSC and Non-CSC Subpopulations

**Figure 5:**
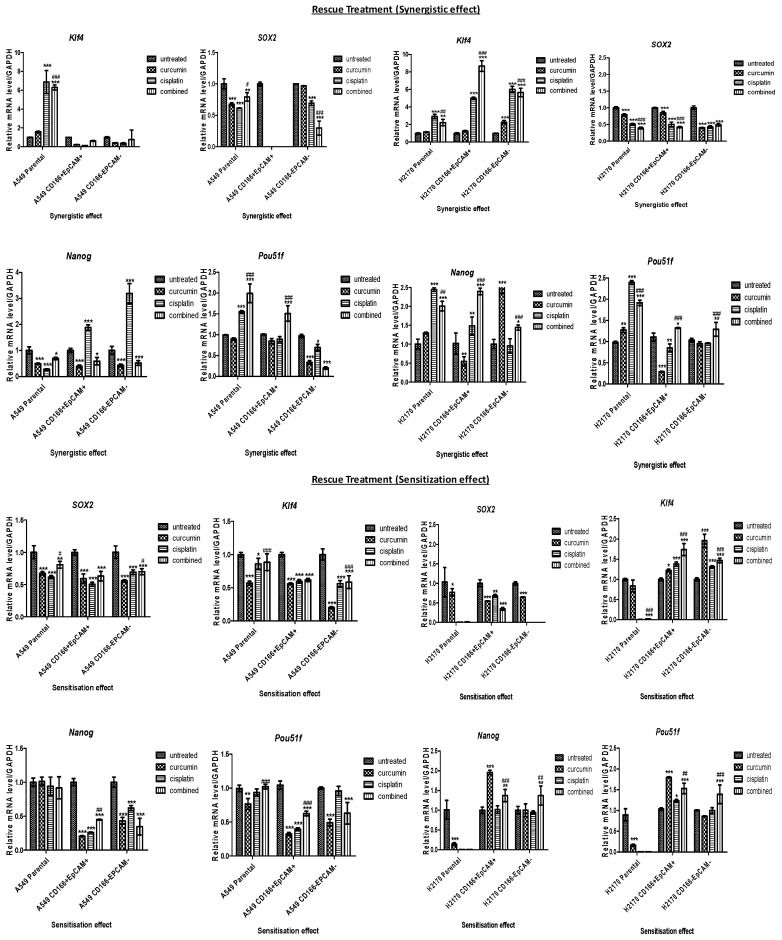
The mRNA expression of stemness genes (SOX2, NANOG, KLF4, and POU51F) after treated with either single (curcumin or cisplatin) or combined treatment, 48 h post-treatment in CD166 + EpCAM + and CD166-EpCAM- non-CSCs subpopulations of A549 (A) and H2170 (B) in both synergistic effect (rescue) and sensitisation (preventive) treatments. Detectable expression levels of the genes were found in all H2170 subpopulations. Each bar represents the average mean ±SD of triplicate samples. Statistical significance was measured with the two-way ANOVA. * *p* < 0.05, ** *p* < 0.01, *** *p* < 0.001 compared with untreated cells. # *p* < 0.05, ## *p* < 0.01, ### *p* < 0.001 compared with curcumin alone.

To understand the mechanism by which self-renewal regulation in the maintenance of CSCs, the expression of stemness-associated genes (*SOX2, NANOG, KLF4*, and *POU51F*) was evaluated (Figure 5). The gene expression level of *SOX2* was down-regulated after treatment with either single or combined treatment in both the CSC and non-CSC subpopulations. In the A549 CD166 + EpCAM + CSC subpopulation, curcumin alone (fold change (FC): −3.005), cisplatin alone (FC: −4.148), and the combination (FC: −3.1095) treatments significantly (*p* < 0.001) suppressed the expression of *SOX2*. NANOG expression was also down-regulated in the curcumin alone and combination treatments (FC: −0.411 and FC: −0.237, respectively), but it was up-regulated in the cisplatin alone treatment (FC: 0.2734). The expression of *KLF4* and *POU51F* did not differ significantly in any of the treatments compared to the untreated group of A549 CD166 + EpCAM + CSCs. However, the expression of *SOX2, NANOG, KLF4,* and *POU51F* were significantly (*p* < 0.001) down-regulated in the combination treatment in A549 CD166-EpCAM– non-CSCs.

### 2.4. Secreted Proteins Present in the CSC and Non-CSC Subpopulations

Due to some constraints, the A549 was the only cell line used in this study. The identification of proteins secreted by the A549 CD166 + EpCAM + CSCs subpopulation is important to understand the proteins involved in regulating the niche of CSCs activity in lung cancer. Peaks Studio version 7 (Bioinformatic Solution, Waterloo, ON, Canada) was used to perform de novo sequencing, and database matching was used to obtain proteins from the whole samples of A549 CD166 + EpCAM + CSCs subpopulation. The false detection rate (FDR) was <1% with significance score −log10P was ≥20 were used for protein acceptance.

In the proteomic study, protein samples were analysed for both untreated and treated groups (curcumin alone, cisplatin alone, and combined). By using the Uniprot database library, there were 90 proteins detected in untreated samples, 47 proteins in samples treated with curcumin, 140 proteins in samples treated with cisplatin, and 114 proteins in samples treated with combined treatment in the A549 CD166 + EpCAM + CSCs subpopulation. All identified proteins were then further confirmed with Database for Annotation, Visualisation and Integrated Discovery (DAVID) software for analysis and after the redundant proteins were excluded, a total of 27, 11, 14, and 30 proteins were detected in untreated, curcumin, cisplatin, and combined treatment groups, respectively. The complete list of each identified proteins in the A549 CD166 + EpCAM + CSCs subpopulation is provided in Suppl 1.

The identification of proteins in non-CSCs was also performed in this study. Identifying proteins in the A549 CD166-EpCAM- non-CSCs subpopulation is necessary to observe the interaction between CSCs and non-CSCs to understand the niche and regulation of CSCs activity, especially in lung cancer. A total of proteins found in untreated, curcumin, cisplatin, and combined treatment groups of A549 CD166-EpCAM- non-CSCs subpopulation were 153, 31, 193, and 88 proteins, respectively. Following validation analysis using DAVID software, the final proteins list in A549 CD166-EpCAM- non-CSCs subpopulation for untreated, curcumin, cisplatin, and combined treatment groups were 33, 7, 44, and 25 proteins, respectively. The complete list of each protein in A549 CD166-EpCAM- non-CSCs subpopulation is provided in Suppl 2.

**Figure 6:**
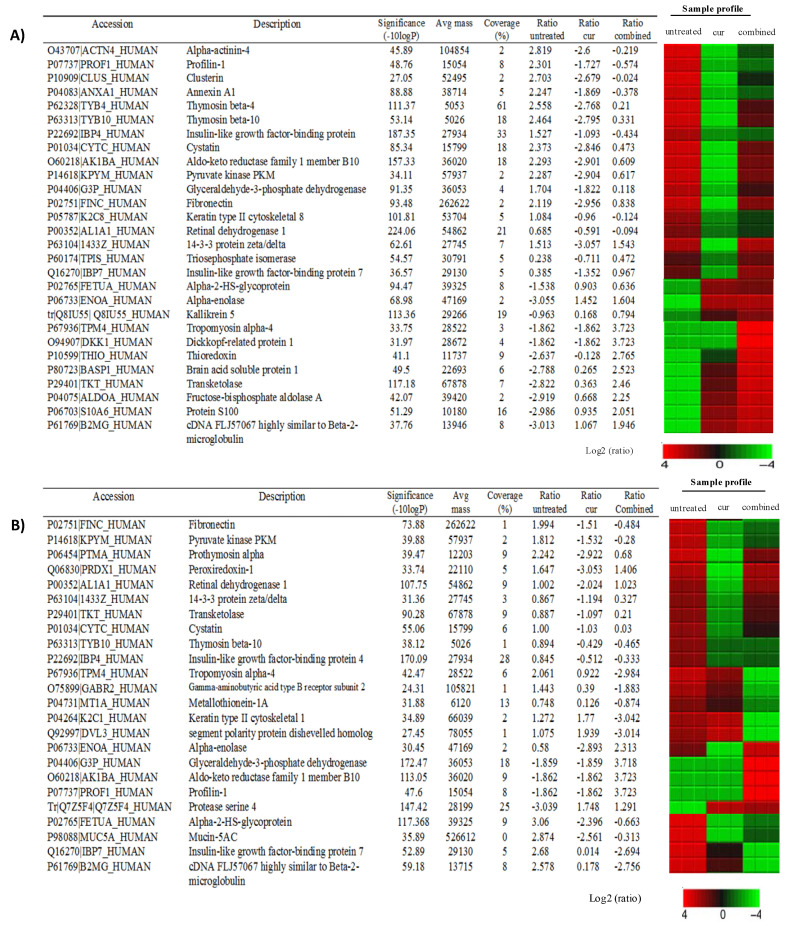
Expression of proteins in CD166 + EpCAM + CSCs (**A**) and CD166-EpCAM-non-CSCs (**B**) subpopulations of A549 cell following treatments with curcumin and in combination with cisplatin as compared to the untreated group. The sample profile in all 12 samples are colour-coded and range from −4 to 4 log.

Secreted proteins that regulate the microenvironment of CSCs and thus could be potential therapeutic targets for the treatment of lung cancer were quantified in this study. The effects of the curcumin and combination treatments were tested for both A549 CD166 + EpCAM + CSC and A549 CD166-EpCAM– non-CSC subpopulations. These treatments were selected to investigate the ability of curcumin to sensitise CSCs to cisplatin. In the A549 CD166 + EpCAM + CSC subpopulation, the proteins of ACTN4, PROF1, CLUS, ANXA1, IBP4, K2C8, and ALDH1A1 exhibited high expression in the untreated group but were down-regulated after treatment with both the curcumin alone and the combination treatment (Figure 6). Several additional proteins (TYB4, TYB10, CYTC, AK1B.4, KPYM, G3P, FINC, 1433Z, TPIS, and IBP7) were down-regulated after treatment with curcumin alone but not with the combination treatment. Additionally, the effect of curcumin alone was much stronger than that of the combination treatment. Other proteins (FETUA, alpha-enolase (ENOA), Q8IU55, TPM4, DKK1, THIO, BASP1, TKT, ALDOA, S10A6, and B2MG) showed low expression in A549 CD166 + EpCAM + CSCs but were up-regulated after treatment with curcumin alone, and the expression of these proteins became stronger after combination treatment.

Many of the proteins (TPM4, GABR2, MT1A, K2C1, DUL3, T1B10, IBP4, IBP7, and B2MG) in the A549 CD166-EpCAM– non-CSC subpopulation were down-regulated after treatment with both curcumin alone and the combination treatment. Other proteins (FINC, KPYM, PTMA, PRDX1, ALHA1, 1433Z, TKT, CYTC, ENOA, FETUA, and MUC5A) were also down-regulated when treated with curcumin alone, but they were not significantly down-regulated in the combination treatment. Only four proteins (G3P, AK1BA, PROF 1, and protease serine 4) were up-regulated after combined treatment compared to curcumin alone (except for protease protein 4 that was up-regulated in the curcumin treatment group)—Figure 6.

DAVID functional annotation had selected only 4 out of 16 pathways in the A549 CD166 + EpCAM + CSC subpopulation, and 3 pathways in the A549 CD166-EpCAM– non-CSC subpopulation (Table 1) that were related to cancer diseases and that involved the proteins YWHAB, YWHAQ, and YWHAZ. Those pathways were the P13/AKT signalling pathway, Hippo signalling pathway, cell cycle, and xenobiotics’ metabolism by cytochrome P450 (Table 1).

## 3. Discussion

The major obstacle in treating cancer is the existence of CSCs, which possess characteristics associated with normal stem cells that make them capable of resist chemo drug treatment and repopulating cancer cells after a certain incubation period. The use of curcumin as the chemo-sensitiser on CSCs was the main objective of the current study. CSCs typically have two phenotypes: actively dividing CSCs and dormant CSCs [18]. Dividing CSCs take part in driving tumour progression, while dormant CSCs ensure resistance to toxic biogenic and repopulate the tumour after chemotherapy. Because actively dividing CSCs have high proliferative activity, most chemotherapy drugs, including cisplatin, are specifically designed to target the actively dividing DNA [19]. In the current study, the A549 CD166 + EpCAM + CSC subpopulation had more proliferative and aggressive properties than the non-CSC subpopulation.

Furthermore, the treatment with curcumin alone was able to reduce the proliferation and eliminated the CSCs in a time-dependent manner. Cisplatin was used as the positive control, as it directly targets the DNA [20] of cells, resulting in a reduction of the CSC subpopulation. The use of cisplatin as a positive control can be seen clearly in targeting cell cycle activities, as shown in the current study (Figure 2). The combination of curcumin and cisplatin provided a greater reduction than treatment with curcumin alone. Thus, curcumin alone and in combination with cisplatin was very effective in inhibiting the A549 CD166 + EpCAM + CSC subpopulation. The cell cycle distribution results showed that the combination treatment significantly arrested the cells as early as at the S-phase, indicating that the treatment is efficient in inhibiting proliferation activity. The decrement of proliferative activity, as seen in A549 cells in the early 24 h up to 48 h and 72 h as depicted in Figure 1, either was due to the induction of apoptosis activity by the single or in combination of treatment as reported earlier by our group [15].

Self-renew is one of the important characteristics used to identify CSCs in the cancer cell population and the normal stem cells as to maintain their stem cell poolsTherefore, targeting self-renewal capability is a crucial strategy in eliminating the CSC population. In this study, the presence of curcumin in the treatment, either with or without a combination with cisplatin was found to reduce not only the number of colonies and spheroid in both A549 and H2170 CD166 + EpCAM + CSC subpopulations, but also the spheroid size. The effect of this treatment was significantly improved when curcumin was combined with cisplatin synergistically. These results show that curcumin can suppress self-renewal activity and inhibit tumour growth. Zhu et al. also reported that curcumin was significantly inhibited the ability of A549 lung CSCs to self-renew, as manifested by suppression of CSC-rich tumorsphere formation [21,22]. A tumour enriched in CSCs would have lower sensitivity to common chemotherapy agents, as reflected by their higher cell viability post-treatment compared to the negative subpopulation. The sensitivity to chemotherapy would be higher due to the existence of non-CSCs in the population [23,24]. Therefore, this study shows that the curcumin has played a significant role in sensitising the CSCs by inhibiting their capability to self-renew

Curcumin has also been reported to possess cancer-preventive properties [25] by suppressing tumour growth and acts as a chemo-preventive agent. Although this study was not conducted in animals, which prevents a definite interpretation, this observation is consistent because curcumin can inhibit CSC activity and down-regulate the stemness genes, as suggested in the in vitro study. In another study, the combination of curcumin and metformin was found to inhibit the self-renewal capability and as a chemo-preventive agent, as shown by the reduction of tumour volume in 4-nitro quinoline-1-oxide (4NQO) induced mice oral carcinogenesis model [26].

The current study results showed that inhibition of self-renewal capability was linked to the down-regulation of *SOX2* and *NANOG* in all CSCs of A549 and H2170 CD166 + EpCAM + CSC subpopulations. The regulation of these genes in CSCs was also reported in other studies [27,28,29,30]

The roles of microenvironment or niche in controlling the stemness of normal and CSCs have been reported in many studies [31,32], which offer a suitable homing site for CSCs. The microenvironment that controls the niche allows CSCs to survive long enough to maintain tumour progression. Bioinformatics analysis revealed that the proteins present in the CSC secretome are those known to play important roles in maintaining the CSC niche/microenvironment. These proteins are involved in chemoresistance, angiogenesis, hypoxia, invasion, metastasis, and proliferation, contributing to sustaining tumour progression.

Persistent expression of aldehyde dehydrogenase (ALDH1A1) in the A549 CD166 + EpCAM + CSC subpopulation for each treatment group indicates its important role resistance of CSCs towards therapy in which some of the cells were found to remain not affected by the treatments given. Due to its specific role in biological activities that eliminate toxic biogenic and xenobiotic aldehydes, this protein is always associated with chemo-resistance [33,34], which leads to the failure of chemotherapy. The expression of AKR1C1, AKR1C2, AKR1C3, and AKR1C4 proteins, which was mainly overexpressed in lung adenocarcinoma (A549) cells [35] and associated with drug resistance in NSCLC [36,37], was found in the A549 CD166 + EpCAM + CSC subpopulation. The study was further support by the expression of AKR1B10 protein, which was reported to promote cell proliferation, growth and induces drug resistance to anti-tumour agents [38,39].

The chemokines/cytokines released by the tumor cells to sustain their microenvironment plays a major role to attract homing process thus induce pre-metastasis [2,40,41,42]. Many proteins, including Annexin A1 (ANXA1), Enolase 1 (ENO1), Insulin-like growth factor-binding protein 4 (IGFBP4), and Cystatin (CST3), are related to the metastasis process. ANXA1 is also known as lipocortin or P35, highly expressed in lung cancer [43], has been shown to attenuate metastasis in breast [44] and prostate-derived cancer-associated fibroblasts [45].

Several signalling pathways that play a crucial role in regulating the lung CSC proteins were found in the A549 CD166 + EpCAM + CSC subpopulation. The pathways detected in the non-CSC subpopulation were similar to those in the CSC subpopulation, likely due to the heterogeneity of the non-CSCs [46]. This heterogeneity is because the non-CSCs are only negative to CD166 and EpCAM cell surface markers, but they might be positive to the other cell surface markers that were not tested in the current study. The YWHAB, YWHAQ, and YWHAZ proteins reported in the current studyare responsible for angiogenesis and migration activities. Harvey et al. reported that ACTB, YWHAB, YWHAQ, and YWHAZ proteins were involved in the Hippo signalling pathway, which controls organ size during development and regeneration [47]. Hippo signalling controls several cellular properties linked to tumorigenesis, including cell proliferation, cell survival, cell resistance, and maintenance of stem cell phenotype [47]. Thus, the YWHAB, YWHAQ, and YWHAZ proteins involved in various biological processes and signalling pathways, could be new targets for future cancer therapy, especially in targeting CSCs in lung cancer.

Using Peaks Studio software, a heat map was produced to analyse the proteins’ fold change after treatment. Curcumin effectively down-regulated the expression of ALDH1A1 and AK1BA (AKR1B10) in the A549 CD166 + EpCAM + CSC subpopulation. This down-regulation indicated that curcumin was able to chemo-sensitise CSCs, thereby increasing their sensitivity to the treatment. This result supports the in vitro data showing that curcumin inhibited CSC activity in both A549 and H2170 cells. The combination treatment also reduced ALDH1A1 expression, but the effect was not as strong as that of curcumin alone.

Curcumin was also found to effectively inhibited TYB10 (TMSB10) and TMSB4 (TYB4), where supressing their expression was reported to inhibit proliferation, migration, and invasion features of cancer cells [48,49]. The persistence regulation of insulin-like growth factor-binding protein (IGFBP), especially IGFBP4 in the current study, demonstrates its important roles as an anti-angiogenic agent. The suppression of anti-angiogenic agents in cancer cells is an important indicator of the inhibitory effect of curcumin in CSC migration, as previously reported by our group [15]. This inhibitory effect of curcumin is similar to that of profilin-1 (Prof-1), although its overexpression in cancer can reduce the tumorigenicity of the cells and inhibit cancer migration, thus indicating that Prof-1 is a tumour suppressor [43,50,51]. Hailing et al. specifically inhibited miR-82 to promote the expression of Prof-1 so that it would suppress proliferation and invasion activity [43]. Several other cancer proteins were up-regulated due to the treatment, including ENOA, TPM4, DKK1, THIO, ALDOA, BASP1, B2MG, S10A6, FETUA, and Q81U55. Most of these proteins take part mainly in the metastasis process. For example, ALDOA was highly expressed in metastatic lung squamous cell carcinoma [52]. Furthermore, its expression is significantly associated with invasion and metastasis in malignant gastric cancer, and it may be involved in glycolysis of CSCs [53]. Likewise, ENOA (ENO1) is known to promote migration and invasion in NSCLC. Some research showed that the up-regulation of ENO1 in NSCLC [53] was associated with proliferation, migration, invasion, and tumorigenicity, although Chang et al. demonstrated that only 26% of ENO1 was expressed NSCLC, it resulted in poor clinical outcome [54]. In another study, overexpression of ENO1 inhibited the epithelial-mesenchymal transition (EMT) in the A549 cell line [55].

The DKK1 protein level was elevated in the A549 CD166 + EpCAM + CSC subpopulation after treatment with both curcumin alone and the combination treatment, which showed that treatment enhanced the expression of DKK1 in these cells. Some research suggests that DKK1 acts as a tumour suppresser by inhibiting cancer cells’ proliferation and metastasis [55]. In the current study, curcumin alone and combination treatment increased DKK1 expression, suggesting the potency of curcumin as a metastasis inhibitor.

The cellular and molecular analyses conducted in this study revealed that combined treatment effectively inhibited the CSC activities than curcumin alone. However, the proteomic analysis indicated that a single treatment with curcumin had a more prominent effect than the combined treatment on disrupting CSC-related proteins. This prominent effect could be because cellular and molecular experiments were designed to focus only on a particular activity, such as cell migration, cell proliferation, or colony or spheroid forming ability, and the combination treatment was very effective in these assays. Although the protein assay only focusing on the A549 subpopulation and is considered to be preliminary data, the proteomic analysis showed the protein profiles of the CSCs niche reveals the treatment itself regulated the proteins that maintain CSCs. While one group of proteins is being suppressed, another group of proteins is activated, and the results vary depending on the treatment. Thus, this finding reflects the orchestrated regulations of CSC niche to maintain their stemness capabilities, and it shows how complex the system of CSCs being regulated. Unfortunately, it was difficult to identify the highly significantly affected proteins by either the single or combination treatment because the protein profiles demonstrated balanced regulation between maintaining the CSC niche and the cytotoxic effect of the treatment.

## 4. Materials and Methods

### 4.1. Cell Culture

NSCLC cell lines of A549 (ATCC^®^ CRL-185™) and H2170 (ATCC^®^ CRL-5928™) were purchased from the American Type Culture Collection (ATCC, Manassas, VA, USA). The A549 and H2170 of CD166 + EpCAM + CSC and CD166EpCAM- non-CSC were isolated as previously described by our group [15,16,17]. The cells were grown in Roswell Park Memorial Institute (RPMI 1640) (Gibco-Life Technologies, Grand Island, NY, USA, Cat. No. 11875101) medium containing 10% foetal bovine serum (FBS) (Gibco-Life Technologies, Cat. No. 16000036) and 1% Penicillin/Streptomycin (PenStrep) (Gibco-Life Technologies, Cat. No. 15-140-122) and grown at 37 °C in a humidified 5% CO_2_ atmosphere. Cells were maintained in T75 tissue culture flasks, and the medium was changed twice a week. Confluent cells were harvested by washing in phosphate-buffered saline (PBS) (Gibco-Life Technologies, Cat. No. 18912014) followed by trypsinisation (0.25% in EDTA (Gibco-Life Technologies, Cat. No. 25300054) for subculture. All cell culture reagents were purchased from Gibco-Life Technologies (Grand Island, NY, USA) unless otherwise stated.

### 4.2. Preparation of Curcumin and Cisplatin

The preparation of curcumin and cisplatin used in this study was previously reported by our group [15]. The various concentrations of curcumin (10, 20, 30, and 40 μM) and cisplatin (5, 10, 15, 20, and 25 μM) were tested on both NSCLC cells for 48 h, and the same concentrations were used in the current study. In brief, both curcumin and cisplatin were purchased from (Sigma-Aldrich, St. Louis, MO, USA, Cat. No. C1386). Curcumin was dissolved in 1 mL Dimethyl sulfoxide (DMSO) to make a stock solution of 10 mM, while cisplatin (Sigma-Aldrich, Cat. No. C2210000) was prepared as a 10 mM stock in 0.9% sodium chloride (NaCl). Both curcumin and cisplatin were then diluted in complete RPMI-1640 medium to provide a substock and final working concentration and were filtered through a 0.22-μm membrane, aliquoted, and stored at −20 °C until further use. The viability of the cell was assessed by the 3-(4,5-dimethylthiazol-2-yl)-2H-tetrazolium, inner salt (MTS) assay by Promega (Madison, WI USA). Cell viability was calculated according to the following formula: Cell viability (%) = cells (sample)/cells (control) × 100 and IC_50_ was calculated using log formula.

### 4.3. Cell Proliferation Assay

Prestoblue (Invitrogen Life Sciences, Paisley, UK, Cat. No. A13261) was used to measure cell proliferation activity. Briefly, cells were seeded at a density of 1 × 10^4^ cells per well in 96-well plates with a final volume of 90 µL RPMI1640 medium, and they were incubated for different periods (24, 48, and 72 h) following treatments with the designated concentration of curcumin, cisplatin and in the combination of both in a humidified 5% CO_2_ incubator at 37 °C. After a specific allocated time point, 10 µL of prestoblue were added to each individual well, and the cells were incubated in a humidified 5% CO_2_ incubator at 37 °C for 20 min to 2 h in the dark. Absorbance in triplicate of 100 µL of each well was measured with a FluorOmega device at 570 nm, using wells without cells as the blank. The viability of cells at different time points was calculated according to the following formula:

Cell viability (%) = Cells (sample)/Cells (control) × 100

### 4.4. Cell Cycle Analysis by Flow Cytometry

Cells were cultured at a density of 4 × 10^5^ cells per well in 6-well plates and grown until they reached approximately 80% confluence. Cells were treated with the half-maximal inhibitory concentration (IC_50_) of curcumin or/and cisplatin for 48 h. After treatment, cells were fixed using ice-cold 70% ethanol applied dropwise and incubated at 4 °C overnight. After incubation, cells were washed twice with cold PBS and then suspended in propidium iodide (PI) (100 µg/mL) and ribonuclease A (20 ng/mL) in PBS for 30 min in a dark room. Cells were analysed using flow cytometry (FACSCalibur instrument, Becton Dickinson BD, Franklin Lakes, NJ, USA) with a cell count of 15,000 cells per sample. Finally, the DNA content of the cells at different phases of the cell cycle was evaluated using ModFit software (Version 3.2, Verity Software House, Topsham, ME, USA). The experiment and analysis were performed in triplicate.

### 4.5. Clonogenic Assay

Cells were seeded in triplicate (1000 cells/well) in 6-well plates. As to determine the inhibitory effect (rescue treatment) of curcumin on self-renewal capability, the colonies were allowed to grow for 7 d. Once colonies were formed, they were treated with either curcumin, cisplatin, or in the combination of both for 48 h before the experiment ended. Meanwhile, preventive treatment was performed by treating the cells with specific treatment at 48 h before the cells were subjected to colonies assay. Afterwards, cells were washed twice with PBS and fixed with 10% formalin for 10 min at room temperature. Giemsa stain (Sigma-Aldrich, Cat. No. G4507) was added (1 mL/well) to the colonies, which were then incubated in the dark or covered using aluminium foil room temperature for approximately 30 min. The plates were then rinsed with distilled water (dh_2_O), and colonies containing over 50 cells were manually counted and photographed using an inverted microscope.

### 4.6. Spheroid Assay

Cells were suspended in serum-free stem cell medium containing Dulbecco’s Modified Eagle Medium (DMEM)/F12K (1:1) (Gibco-Life Technologies, Cat. No. 11320033) supplemented with 10 ng/mL basic fibroblast growth factor (bFGF) (Gibco-Life Technologies, Cat. No. 13256-029), 1% B27 (Gibco-Life Technologies, Cat. No. 17504044), 20 ng/mL epidermal growth factor (EGF) (Gibco-Life Technologies, Cat. No. PHG0313), and 1% antibiotic-antimycotic (PenStrep). The cells were re-suspended at a ratio of 1:10 (*v*/*v*) of growth factor-reduced Matrigel (BD Biosciences, San Jose, CA, USA, Cat. No: 356231) in serum-free sphere medium. The cells (200 cells/well) were then plated in ultra-low attachment dishes. As to determine the inhibition effect (rescue treatment) of curcumin on self-renewal capability, cells were treated with specific treatments on day 7 after the formation of spheroids. In the preventive treatment, cells were treated at 48 h after the seeding. Spheroid formation was assessed by light microscopy (Olympus Tokyo, Japan) after 21 day of culture. All growth factors were purchased from Life Technologies (Carlsbad, CA, USA).

### 4.7. Quantitative Real-Time-Polymerase Chain Reaction (qRT-PCR)

To evaluate the effect of the treatment on the stemness capability of CSCs, the total RNA wasextracted using an RNA easy extraction kit (Qiagen, Hamburg, Germany, Cat. No. 74106) according to the manufacturer’s protocol. cDNA was then synthesised from 1 µg of total RNA using the Tetro cDNA synthesis Kit (Bioline, London, UK, Cat. No. BIO-65043). qRT-PCR was performed using an ABI StepOnePlus™ PCR System (Applied Biosystems, Foster City, CA, USA). Taqman probe as to evaluate the expression of transcription factor genes of SOX2, POU51F, NANOG, KLF4, and housekeeping gene, the GAPDH gene was purchased from First Base (Singapore) Table 2. The qRT-PCR reaction was prepared using a SensiFAST Probe Hi-ROX Kit (Bioline, Cat. No. BIO82005). The RT-qPCR reactions were run under the following cycle conditions: 50 °C for 2 min, 95 °C for 20 s, 40 cycles at 95 °C for 15 s, and 60 °C for 1 min. The basic relative gene expression was calculated using the 2^–ΔΔCt^ formula, and results were normalised to the endogenous control (housekeeping gene, GAPDH). All experiments were conducted in triplicate.

### 4.8. Preparation of Condition Medium

As to evaluate the effect of the treatments on the microenvironment/niche of the CSCs, the protein released by the treated cells will be collected and evaluated. To achieve that CSCs were cultured in RPMI 1640 supplemented with 10% FBS at 37 °C in a humidified incubator with 5% CO_2_. Cells were cultured in tissue T75 culture flasks at a density of 1 × 10^6^ cells/flask and grown until they reached 80–90% confluence. Cells were then washed three times with PBS to remove the excess FBS and subsequently incubated with serum-free medium for 48 h. For treatment, cells were incubated either with a single treatment (curcumin or cisplatin) or combined treatment in a serum-free medium for 48 h. The conditioned medium was then collected and centrifuged (400 *g*, 10 min) to eliminate the intact cells, filtered with a 0.2 µm filter, and lyophilised using a freeze dryer (Alpha 1-2 LDplus, Christ, Osterode am Harz, Germany). Each treatment was tested in five independent replicates, and replicates were pooled before being lyophilised.

### 4.9. Trichloroacetic Acid (TCA) Precipitation

Due to some constraints, only A549 cell lines were used in protein assay. To evaluate the proteins released by the CSCs into the microenvironment/niche, the lyophilised samples were dissolved in 1 mL double dh_2_O and were subjected to TCA precipitation (TCA/ddh_2_O = 1:4) followed by 90 min of incubation at 4 °C. Samples were then centrifuged using a fixed rotor (10,000 *g*, 4 °C, 30 min). The supernatant was discarded, and 2 mL of chilled acetone was added to the samples, followed by incubation for 15 min at 4 °C. The protein samples were centrifuged again (10,000 *g*, 4 °C, 15 min), and the supernatant was discarded. Finally, ~30–50 µL of ddh_2_O were added to the samples and used to measure protein concentrations.

### 4.10. Micro BCA Assay

Total protein quantification was carried out according to the Micro BCA Protein Assay Kit (Thermo Fisher Scientific, San Jose, CA, USA, Cat. No. 23235) protocol. A 150 µL aliquot of each standard (bovine serum albumin (BSA)) or sample was pipetted into a 96-well plate. The 150 µL quantity of working reagents (A, B, and C) was added to each well, and the plate was gently shaken for 30 s and then incubated at 37 °C for 2 h. A standard curve ranging from 0 to 200 µg was constructed using BSA with 562 nm absorbance. The total protein concentration was calculated by subtracting the absorbance reading of the blank standard (0 µg) to absorb the sample’s absorbance reading.

### 4.11. In-Solution Protein Digestion

Samples were resuspended in 100 µL of 6 M urea and 100 mM tris buffer containing 1 mg of total protein. Briefly, 5 µL of 200 nM DTT (reducing agent) (Bio-Rad laboratories, Hercules, Ca, USA, Cat. No. 1610611) was added to each sample, kept at room temperature for 1 h. Next, 20 µL of 200 mM of iodoacetamide (alkylating agent) were added, and the incubation was continued at room temperature for 1 h, followed by the addition of another 20 µL of 200 mM iodoacetamide (Bio-Rad laboratories, Cat. No. 1632109) for another 1 h. Urea concentration was then reduced to ~0.6 M by adding 775 µL of water (a concentration at which trypsin retains its activity). Finally, digestion was performed by adding 20 µg of trypsin (Promega, Fitchburg, WI, USA, Cat. No. V5820) solution to each sample followed by incubation overnight (≤16 h) at 37 °C. The digestion was stopped on the next day by adjusting the pH solution to pH < 6 using concentrated acetic acid.

### 4.12. Liquid Chromatography-Mass Spectrometry (LC-MS/MS) Analysis

Each of the samples was mixed with 100 µL of 0.1% formic acid in deionised water and filtered using a 0.45 µm regenerated cellulose membrane syringe filter (Sartorius AG, Goettingen, Germany). A Linear Trap Quadropole (LTQ)-orbitrap Velos pro-mass spectrometer coupled with an Easy-nLC II nano liquid chromatography system was used for proteomic analysis. Easy column C18 (10 cm, 0.75 mm i.d., 3 µm) was used as the analytical column, and Easy column C18 (2 cm, 0.1 mm i.d., 5 µm) was used as the pre-column (Thermo Scientific, San Jose, CA, USA). The pre-column was equilibrated at a flow rate of 3 µL/min for 15 µL, and the analytical column was equilibrated at a flow rate of 0.3 µL /min for 4 µL. Three microliters of a sample were injected and chromatographically separated at a flow rate of 0.3 µL/min. Running buffers used were: (A) 0.1% formic acid in deionised water and (B) 0.1% formic acid in acetonitrile. Samples were eluted using the gradient 5% to 100% of buffer B in 80 min. The eluent was sprayed into the mass spectrometer at 2.1 kV 9 source voltages, and the temperature was set at 220 °C. Peptides were detected by full scan mass analysis from m/z 300 to 2000 at resolving power of 60,000 (at m/z 400, FWHM; 1 s acquisition) with data-dependent MS/MS analysis (ITMS) triggered by the eight most abundant ions from the parent mass list of predicted peptides with rejection of singly or unassigned charge state. Collision-induced dissociation was applied at the fragmentation technique with a collision of 35.

### 4.13. Protein Identification and Analysis

Peaks Studio Version 7 (Bioinformatics Solutions, Waterloo, ON, Canada) was used to perform de novo sequencing and database matching. Carbamidomethylation and methionine oxidation were set as fixed modifications, and maximum missed cleavage was set at 2. The Uniprot Homo sapiens database from March 2013 was used for database matching. The de novo sequencing parameter for parent mass and precursor mass tolerance was set at 0.1 Da, while fragment mass error tolerance was set at 0.8 Da. A false detection rate of <1% and significance score (–10 lgP) for protein >20 were used for protein acceptance. A minimum unique peptide was set at 1.

### 4.14. Functional Group Analysis

The proteins obtained using Peaks Studio Version 7 were assigned into functional categories using Panther Classification System (http://pantherdb.org) and DAVID (https://david.ncifcrf.gov).

### 4.15. Statistical Analysis

All data were expressed as the mean ± standard deviation (SD) of three independent experiments. Two-way analysis of variance was performed using Graph Pad Prism software (GraphPad Software Inc., San Diego, CA, USA; graphpadprism.software.informer.com/6.0) to identify significant differences among the treatment groups for in vitro assays. *p* < 0.05 was considered to be statistically significant.

## 5. Conclusions

Cancer treatment has shown tremendous improvement over the years despite variation in terms of achievement due to its complexity to treat. The ability to identify and characterise CSCs had opened a new hope for a better strategy in cancer therapy. The ability of CSCs to resist chemotherapeutic drugs has led CSCs to become a central focus point because of their ability to repopulate the cancer cells and escapes therapeutic drugs, thus metastasis to other parts of the organs. The results of the current study revealed that cucurmin has its own degree in supressing the self-renewal capability of CSCs population and sensitising them towards chemotherapy, thus could lead to an effective therapeutic target.

The cancer niche plays an important role in maintaining CSC population [32]. The understanding the active components, especially on the protein regulation that constitutes the niche or microenvironment of CSCs either due to secreted by the CSCs themselves or cells surround the CSCs where the secreting compounds as part of the cellular protection from further damaged due to CSCs, is greatly important for future specific targeted protein to treat lung cancer. By designing the in vitro study carried out in this study, one could eliminate the hypothesis in which the secreted proteins are solely by the CSC and not by the normal cells as part of their protective mechanisms against foreign cells. Targeting multiple pathways and proteins responsible for CSC regulation may eliminate CSC subpopulations. In this study, proteomic analysis had identified several pathways that involved in cancer such as the P13/AKT, hippo signalling, and cell cycle pathways that are directly involved in cancer progression via cell proliferation, invasion, and metastasis, thus they could be targeted for developing cancer therapeutics. Furthermore, these three pathways contain the same proteins (YWHAB, YWHAQ, and YWHAZ), suggesting that they could be critical therapeutic target proteins. The involvement of these proteins in therapeutic resistance needs to evaluate further. Therefore, results of this study show that either alone or in combination with cisplatin, curcumin can suppress CSC properties; thus, it could be an effective therapeutic strategy to prevent the emergence of chemoresistance in NSCLC by eliminating CSCs.

## Figures and Tables

**Table 1:** KEGG pathways related to CD166+EpCAM+ CSCs subpopulation and CD166-EPCAM- non- CSCs subpopulation of A549 cell line.

Group	KEGG Pathway	Genes	%	*p*-Value	Benjamin
**A549 CD166-EPCAM-CSCs** **SUBPOPULATION**	Steroid hormone biosynthesis	4	15.4	4.2 × 10^−4^	2.3 × 10^−2^
	Glycolysis	4	15.4	6.4 × 10^−4^	1.8 × 10^−2^
	Biosynthesis of amino acids	4	15.4	8.6 × 10^−4^	1.6 × 10^−2^
	Carbon metabolism	4	15.4	2.9 × 10^−3^	4.0 × 10^−2^
	Fructose and mannose metabolism	3	11.5	3.0 × 10^−3^	3.3 × 10^−2^
	* Hippo signalling pathway	4	15.4	6.6 × 10^−3^	6.0 × 10^−2^
	* Metabolism of xenobiotics by cytochrome P450	3	11.5	1.5 × 10^−2^	1.2 × 10^−1^
	Biosynthesis of antibiotics	4	15.4	1.7 × 10^−2^	1.1 × 10^−1^
	Metabolic pathways	8	30.8	2.8 × 10^−2^	1.6 × 10^−1^
	Oocyte meiosis	3	11.5	3.2 × 10^−2^	1.7 × 10^−1^
	* Cell cycle	3	11.5	4.1 × 10^−2^	1.9 × 10^−1^
	Hepatitis B	3	11.5	5.4 × 10^−2^	2.3 × 10^−2^
	* PI3-AKT signalling pathway	4	15.4	5.8 × 10^−2^	2.3 × 10^−1^
	Influenza A	3	11.5	7.4 × 10^−2^	2.7 × 10^−1^
	Epstein-Barr virus infection	3	11.5	8.6 × 10^−2^	2.9 × 10^−1^
	Viral carcinogenesis	3	11.5	9.8 × 10^−2^	3.0 × 10^−1^
**A549 CD166-EPCAM-NON-CSCs** **SUBPOPULATION**	* Hippo signalling pathway	8	24.2	7.2 × 10^−7^	4.5 × 10^−4^
	* Cell cycle	7	21.2	4.06 × 10^−6^	1.2 × 10^−4^
	Oocyte meiosis	6	18.2	3.7 × 10^−5^	7.7 × 10^−4^
	Epstein-Barr virus infection	7	21.2	4.6 × 10^−5^	7.2 × 10^−4^
	Viral carcinogenesis	7	21.2	7.0 × 10^−5^	8.8 × 10^−4^
	* PI3-AKT signalling pathway	7	21.2	1.2 × 10^−3^	1.2 × 10^−2^
	Hypertrophic cardiomyopathy (HCM)	4	12.1	2.7 × 10^−3^	2.4 × 10^−2^
	Dilated cardiomyopathy	4	12.1	3.3 × 10^−3^	2.6 × 10^−2^
	Regulation of actin cytoskeleton	5	15.2	6.5 × 10^−3^	4.4 × 10^−2^
	Shigellosis	3	9.1	2.2 × 10^−2^	1.3 × 10^−1^
	Bacterial invasion of epithelial cells	3	9.1	3.2 × 10^−2^	1.7 × 10^−1^
	Salmonella infection	3	9.1	3.6 × 10^−2^	1.7 × 10^−1^
	Hepatitis B	3	9.1	9.6 × 10^−2^	3.9 × 10^−1^

* Pathways relevant to cancer and non-CSCs regulation.

**Table 2:** List of Taqman^®^ Gene Expression Probes.

Accession Number	Gene Symbol	Amplicon Length
Hs01053049_s1	*SOX2*	91
Hs00999632_g1	*POU51F*	77
Hs04399610_g1	*NANOG*	101
Hs00358836_m1	*KLF4*	110
Hs02758991_g1	*GAPDH*	93

## Supplementary Materials

The following are available online.
