# Supplementary material for: Synergistic Roles of Curcumin in Sensitising the Cisplatin Effect on a Cancer Stem Cell-Like Population Derived from Non-Small Cell Lung Cancer Cell Lines
Source: Molecules. 2021 Feb 18;26(4):1056. doi: 10.3390/molecules26041056 (PMC7922800; doi:10.3390/molecules26041056)
Supplement: Supplementary file 1 [file molecules-26-01056-s001.pdf]

Supplementary I

List of detected proteins in A549 CD166+EpCAM+ CSCs subpopulation derived from NSCLC, untreated sample.

Uniprot accession	Protein name/Description	Gene symbol	-10lgP	Coverage (%)	#Peptides	#Unique
S10A6_HUMAN	S100 calcium binding protein A6	S1006	51.29	16	1	1
Q1KLZ0_HUMAN	Actin beta	ACTB	160.32	19	5	5
AL1A1_HUMAN	Aldehyde dehydrogenase 1 family member A1	ALDH1A1	2224.06	21	7	7
AK1BA_HUMAN	Aldo-keto reductase family 1 member B10	AKR1B10	157.33	18	4	4
AK1C1_HUMAN	Aldo-keto reductase family 1 member C1	AKR1C1	128.53	16	4	1
AK1C2_HUMAN	Aldo-keto reductase family 1 member C2	AKR1C2	131.3	16	4	1
AK1C3_HUMAN	Aldo-keto reductase family 1 member C3	AKR1C3	128.53	16	4	1
AK1C4_HUMAN	Aldo-keto reductase family 1 member C4	AKR1C4	128.53	16	4	1
ALDOA_HUMAN	Aldolase, fructose-bisphosphate A	ALDOA	42.07	2	1	1
FETUA_HUMAN	Alpha 2-HS glycoprotein	AHSG	94.47	8	3	3
ANXA1_HUMAN	Annexin A1	ANXA1	88.88	5	1	1
H0YC35_HUMAN	Clusterin 1	CLU	27.05	3	1	1
CYTC_HUMAN	Cystatin C	CST3	85.34	18	2	2
K7EM90_HUMAN	Enolase 1	ENO1	68.98	5	1	1
B7ZLE5_HUMAN	Fibronectin 1	FN1	93.48	2	4	4
G3P_HUMAN	Glyceraldehyde-3-phosphate dehydrogenase	GAPDH	91.35	4	1	1
A0A5E8_HUMAN	Growth arrest specific 2 like 1	GAS2L1	27.64	1	1	1
IBP4_HUMAN	Insulin like growth factor binding protein 4	IGFBP4	187.35	33	8	8
Q7L4M3_HUMAN	Keratin8	KRT8	101.81	8	2	2
Q3SY19_HUMAN	Protease, serine1	PRSS1	69.6	17	3	2

Suppl 1: Continued

Uniprot accession	Protein name/Description	Gene symbol	-10lgP	Coverage (%)	#Peptides	#Unique
Q7Z5F4_HUMAN	Protease, serine3	PRSS3	127.42	26	7	4
D6W5K2_HUMAN	Thymosin beta 10	TMSB10	53.14	5	1	1
A2VCK8_HUMAN	Thymosin beta 4, X-linked	TMSB4X	111.37	61	2	2
TPIS_HUMAN	Triosephosphate isomerase 1	TP1	54.57	5	1	1
B5BU24_HUMAN	Tyrosine 3-monooxygegenase/tryptophan 5-monooxygenase activation protein beta	YWHAB	62.61	7	2	7
1433T_HUMAN	Tyrosine 3-monooxygegenase/tryptophan 5-monooxygenase activation protein theta	YWHAQ	62.61	7	2	7
E7EX29_HUMAN	Tyrosine 3-monooxygegenase/tryptophan 5-monooxygenase activation protein zeta	YWHAZ	62.61	7	2	2

List of detected proteins in A549 CD166+EpCAM+ CSCs subpopulation derived from NSCLC, curcumin sample.

Uniprot accession	Protein name/Description	Gene symbol	-10lgP	Coverage (%)	#Peptides	#Unique
H0YMG7_HUMAN	Aldehyde dehydrogenase 1 family member A2	ALDH1A2	30.9	2	1	1
Q7Z3A2_HUMAN	Aldehyde dehydrogenase 1 family member A3	ALDH1A3	30.9	2	1	1
A8MYB8_HUMAN	Aldehyde dehydrogenase 3 family member A1	ALDH3A1	30.9	2	1	1
J3QRD1_HUMAN	Aldehyde dehydrogenase 3 family member A2	ALDH3A2	30.9	2	1	1
E9PJV0_HUMAN	Aldehyde dehydrogenase 3 family member B2	ALDH3B2	30.9	5	1	1
FETUA_HUMAN	Alpha 2-HS glycoprotein	AHSG	112.03	21	5	5
H0YLF3_HUMAN	Beta-2-microglobulin	B2M	37.76	14	1	1
IBP4_HUMAN	Insulin like growth factor binding protein 4	IGFBP4	95.11	12	2	2
TRY6_HUMAN	Protease, serine3 pseudogene 2	PRSS3P2	70.1	16	3	2
Q7Z5F4_HUMAN	Protease, serine3	PRSS3	157.22	28	15	8
THIO_HUMAN	Thioredoxin	TXN	41.1	9	1	1

List of detected proteins in A549 CD166+EpCAM+ CSCs subpopulation derived from NSCLC, cisplatin sample.

Uniprot accession	Protein name/Description	Gene symbol	-10lgP	Coverage (%)	#Peptides	#Unique
S10A6_HUMAN	S100 calcium binding protein A6	S1006	40.05	16	1	1
Q1KLZ0_HUMAN	Actin beta	ACTB	160.36	26	6	6
ACTG_HUMAN	Actin gamma 1	ACTG1	160.36	26	6	6
ALBU_HUMAN	Albumin	ALB	130.34	8	4	4
AL1A1_HUMAN	Aldehyde dehydrogenase 1 family member A1	ALDH1A1	162.23	16	7	7
AK1BA_HUMAN	Aldo-keto reductase family 1 member B10	AKR1B10	69.28	8	3	3
FETUA_HUMAN	Alpha 2-HS glycoprotein	AHSG	65.85	6	2	2
B2MG_HUMAN	Beta-2-microglobulin	B2M	59.44	8	1	1
BASP1_HUMAN	Brain abundant membrane attached signal protein 1	BASP1	54.47	6	1	1
E7ERK6_HUMAN	Clusterin 1	CLU	63.61	6	1	1
CYTC_HUMAN	Cystatin C	CST3	51.32	6	1	1
NEUA_HUMAN	Cytidine monophosphate N-acetylneuraminic acid synthetase	CMAS	38.92	2	1	1
A4QMW8_HUMAN	Enolase 1	ENO1	36.6	6	1	1
EF1A3_HUMAN	Eukaryotic translation elongation factor 1 alpha pseudogene 5	EEF1A1P5	45.13	2	1	1
Q6IPS9_HUMAN	Eukaryotic translation elongation factor 1 alpha 1	EEF1A1	45.13	2	1	1
EF1A2_HUMAN	Eukaryotic translation elongation factor 1 alpha 2	EEF1A2	45.13	9	1	1
Q6N084_HUMAN	Fibronectin 1	FN1	26.1	1	1	1
GABR2_HUMAN	Gamma-aminobutyric acid type V receptor subunit 2	GABBR2	28.15	1	1	1
G3P_HUMAN	Glyceraldehyde-3-phosphate dehydrogenase	GAPDH	38.59	2	1	1
Q6PK50_HUMAN	Heat shock protein 90 alpha family class B member 1	HSP90AB1	32.41	3	1	1

Continued

Uniprot accession	Protein name/Description	Gene symbol	-10lgP	Coverage (%)	#Peptides	#Unique
H90B3_HUMAN	Heat shock protein 90 alpha family class B member 3, pseudogene	HSP90AB3P	32.41	2	1	1
IBP4_HUMAN	Insulin like growth factor binding protein 4	IGFBP4	144.5	17	3	3
IBP7_HUMAN	Insulin like growth factor binding protein 7	IGFBP7	36.57	5	1	1
K2C8_HUMAN	Keratin8	KRT8	61.95	2	1	1
K7EQQ3_HUMAN	Keratin9	KRT9	85.84	6	1	1
TRY6_HUMAN	Protease, serine3 pseudogene 2	PRSS3P2	75.69	10	4	2
Q7Z5F4_HUMAN	Protease, serine3	PRSS3	136.7	19	9	4
KPYM_HUMAN	Pyruvate kinase, muscle	PKM	34.11	2	1	1
THIO_HUMAN	Thioredoxin	TXN	41.97	9	1	1
D6W5K2_HUMAN	Thymosin beta 10	TMSB10	26.68	5	1	1
PIS_HUMAN	Triosephosphate isomerase 1	TPI1	80.97	5	1	1
Q5VU62_HUMAN	Tropomyosin 3	TPM3	28.73	5	1	1
K7ERG3_HUMAN	Tropomyosin 4	TPM4	28.73	5	1	1
E7EX29_HUMAN	Tyrosine 3-monooxygegenase/tryptophan 5-monooxygenase activation protein zeta	YWHAZ	98.32	15	3	3

List of detected proteins in A549 CD166+EpCAM+ CSCs subpopulation derived from NSCLC, combined treatment sample.

Uniprot accession	Protein (David)	Gene symbol (David)	-10lgP	Coverage (%)	#Peptides	#Unique
C9JXI9_HUMAN	KRIT1, ankyrin repeat containing	KRIT1	24.54	24	1	1
S10A6_HUMAN	S100 calcium binding protein A6	S1006	67.78	16	1	1
Q1KLZ0_HUMAN	Actin beta	ACTB	41.49	3	1	1
ACTN4_HUMAN	Actinin alpha 4	ACTN4	45.89	2	2	2
AL1A1_HUMAN	Aldehyde dehydrogenase 1 family member A1	ALDH1A1	153.26	13	6	6
AK1BA_HUMAN	Aldo-keto reductase family 1 member B10	AKR1B10	91.61	9	2	2
ALDOA_HUMAN	Aldolase,fructose-bisphosphate A	ALDOA	53.22	6	2	2
FETUA_HUMAN	Alpha 2-HS glycoprotein	AHSG	115.57	8	4	4
B2MG_HUMAN	Beta-2-microglobulin	B2M	58.95	8	1	1
BASP1_HUMAN	Brain abundant membrane attached signal protein 1	BASP1	49.5	6	1	1
CYTC_HUMAN	Cystatin C	CST3	51.34	6	1	1
NEUA_HUMAN	Cytidine monophosphate N-acetylneuraminic acid synthetase	CMAS	33.51	2	1	1
DKK1_HUMAN	Dickkopf WNT signaling pathway inhibitor 1	DKK1	31.97	4	1	1
A4QMW8_HUMAN	Enolase 1	ENO1	34.04	6	1	1
GABR2_HUMAN	Gamma-aminobutyric acid type V receptor subunit 2	GABBR2	25.44	1	1	1
G3P_HUMAN	Glyceraldehyde-3-phosphate dehydrogenase	GAPDH	62.31	9	3	3
IPIL1_HUMAN	Inositol1, 4, 5-triphosphate receptor interacting protein-like 1	ITPRIPL1	28.09	2	1	1

Continued

Uniprot accession	Protein (David)	Gene symbol (David)	-10lgP	Coverage (%)	#Peptides	#Unique
IBP4_HUMAN	Insulin like growth factor binding protein 4	IGFBP4	155.81	20	4	4
IBP7_HUMAN	Insulin like growth factor binding protein 7	IGFBP7	42.96	5	1	1
PPIA_HUMAN	Peptidylproly isomerase A	PPIA	52.07	7	1	1
PROF1_HUMAN	Profilin 1	PFN1	48.76	8	1	1
Q8N2U3_HUMAN	Protease, serine3	PRSS3	131.73	31	14	1
KPYM_HUMAN	Pyruvate kinase, muscle	PKM	32.16	2	1	1
THIO_HUMAN	Thioredoxin	TXN	46.63	9	1	1
D6W5K2_HUMAN	Thymosin beta 10	TMSB10	44.2	8	2	2
TKT_HUMAN	Transketolase	TKT	117.18	7	3	3
TPIS_HUMAN	Triosephosphate isomerase 1	TPI1	102.23	15	2	2
Q5VU62_HUMAN	Tropomyosin 3	TPM3	33.75	5	1	1
K7ERG3_HUMAN	Tropomyosin 4	TPM4	33.75	5	1	1
E7EX29_HUMAN	Tyrosine 3-monooxygegenase/tryptophan 5-monooxygenase activation protein zeta	YWHAZ	112.71	15	3	1

Supplementary II

List of detected proteins in A549 CD166-EpCAM- non-CSCs subpopulation derived from NSCLC, untreated sample.

Uniprot accession	Protein name/Description	Gene symbol	-10lgP	Coverage (%)	#Peptides	#Unique
Q7Z5F4_HUMAN	Protease, serine 3	PRSS3	104.71	24	8	4
TRY6_HUMAN	Protease, serine 3 pseudogene 2	PRSS3P2	64.85	19	4	2
C9JFL5_HUMAN	Actin, gamma 2, smooth muscle, enteric	ACTG2	87.53	16	2	2
J3KT65_HUMAN	Actin gamma 1	ACTG1	87.53	12	2	2
CYTC_HUMAN	Cystatin C	CST3	55.06	6	1	1
GABR2_HUMAN	Gamma-aminobutyric acid type B receptor subunit 2	GABBR2	24.31	1	1	1
FINC_HUMAN	Fibronectin 1	FN1	73.88	1	3	3
FETUA_HUMAN	Alpha 2-HS glycoprotein	AHSG	117.68	6	3	3
IBP7_HUMAN	Insulin like growth factor binding protein 7	IGFBP7	52.89	5	1	1
IBP4_HUMAN	Insulin like growth factor binding protein 4	IGFBP4	170.09	28	6	6
F6QUT6_HUMAN	Actin, alpha 2, smooth muscle, aorta	ACTA2	87.53	15	2	2
Q14887_HUMAN	Mucin 5AC, oligomeric mucus/gel-forming	MUC5AC	35.89	1	1	1
H0YLF3_HUMAN	Beta-2-microglobulin	B2M	59.18	14	1	1
K7EJ44_HUMAN	Profilin 1	PFN1	47.6	11	1	1
1433S_HUMAN	Stratifin	SFN	31.36	3	1	1
MT1A_HUMAN	Metallothionein 1A	MT1A	31.88	13	1	1
A6NL76_HUMAN	Actin, alpha 1, skeletal muscle	ACTA1	87.53	8	2	2
NEUA_HUMAN	Cytidine monophosphate N-acetylneuraminic acid synthetase	CMAS	34.49	1	1	1
AL1A1_HUMAN	Aldehyde dehydrogenase 1 family member A1	ALDH1A	107.75	9	5	5
K7ERG3_HUMAN	Tropomyosin 4	TPM4	42.47	9	2	2
Q5VU62_HUMAN	Tropomyosin 3	TPM3	42.47	9	2	2

Continued

Uniprot accession	Protein name/Description	Gene symbol	-10lgP	Coverage (%)	#Peptides	#Unique
A4QMW8_HUMAN	Enolase 1	ENO1	30.45	6	1	1
E7EVS6_HUMAN	Actin beta	ACTB	87.53	14	2	2
KPYM_HUMAN	Pyruvate kinase, muscle	PKM	39.88	2	1	1
Q15254_HUMAN	Prothymosin, alpha	PTMA	39.47	9	1	1
D6W5K2_HUMAN	Thymosin beta 10	TMSB10	38.12	5	1	1
TYB4_HUMAN	Thymosin beta 4, X-linked	TMSB4X	34.03	27	1	1
1433E_HUMAN	Tyrosine 3-monooxygenase/tryptophan 5-monooxygenase activation protein epsilon	YWHAE	31.36	3	1	1
E9PG15_HUMAN	Tyrosine 3-monooxygenase/tryptophan 5-monooxygenase activation protein theta	YWHAQ	31.36	5	1	1
Q4VY20_HUMAN	Tyrosine 3-monooxygenase/tryptophan 5-monooxygenase activation protein beta	YWHAB	31.36	9	1	1
F8WEB6_HUMAN	Tyrosine 3-monooxygenase/tryptophan 5-monooxygenase activation protein eta	YWHAH	31.36	18	1	1
E7EVZ2_HUMAN	Tyrosine 3-monooxygenase/tryptophan 5-monooxygenase activation protein zeta	YWHAZ	31.36	7	1	1
1433G_HUMAN	Tyrosine 3-monooxygenase/tryptophan 5-monooxygenase activation protein gamma	YWHAG	31.36	3	1	1

List of detected proteins in A549 CD166-EpCAM- non-CSCs subpopulation derived from NSCLC, curcumin sample.

Uniprot accession	Protein name/Description	Gene symbol	-10lgP	Coverage (%)	#Peptides	#Unique
THIO_HUMAN	Thioredoxin	TXN	50.93	9	1	1
H6VRG1_HUMAN	Keratin 1	KRT1	34.89	2	1	1
ALBU_HUMAN	Albumin	ALB	109.06	8	4	4
A6XGL3_HUMAN	Protease, serine 1	PRSS1	115.28	29	7	2
Q5NV56_HUMAN	Protease, serine 2	PRSS2	53.02	6	2	2
Q7Z5F4_HUMAN	Protease, serine 3	PRSS3	147.42	25	12	5
CYTC_HUMAN	Cystatin C	CST3	41.38	6	1	1

List of detected proteins in A549 CD166-EpCAM- non-CSCs subpopulation derived from NSCLC, cisplatin sample.

Uniprot accession	Protein name/Description	Gene symbol	-10lgP	Coverage (%)	#Peptides	#Unique
E2DRY6_HUMAN	Enolase 1	ENO1	31.44	2	1	1
TPIS_HUMAN	Triosephosphate isomerase 1	TPI1	75.74	5	1	1
PPIH_HUMAN	Peptidylprolyl isomerase H	PPIH	43.92	7	2	2
FINC_HUMAN	Fibronectin 1	FN1	45.07	0	1	1
DOP1_HUMAN	Dopey family member 1	DOPEY1	27.45	0	1	1
GABR2_HUMAN	Gamma-aminobutyric acid type GABBR2B receptor subunit 2x	GABBR2	24.85	1	1	1
Q3SY19_HUMAN	Protease, serine 1	PRSS1	48.07	6	2	2
A6XMV9_HUMAN	Protease, serine 2	PRSS2	48.07	5	2	2
TRY3_HUMAN	Protease, serine 3	PRSS3	109.96	14	6	4
TRY6_HUMAN	Protease, serine 3 pseudogene 2	PRSS3P2	52.31	6	2	2
H0YAL9_HUMAN	Transmembrane protease, serine 11A	TMPRSS11A	32.05	2	1	1
Q5TA12_HUMAN	Dopey family member 1	DOPEY1	27.45	0	1	1
I1W660_HUMAN	Dickkopf WNT signaling pathway inhibitor 1	DKK1	54.64	4	1	1
DVL3_HUMAN	Dishevelled segment polarity protein 3	DVL3	27.45	1	1	1
I3L2N2_HUMAN	Dishevelled segment polarity protein 2	DVL2	27.45	1	1	1
G3V1A4_HUMAN	Cofilin 1	CFL1	57.38	10	2	2
B4DNK4_HUMAN	Pyruvate kinase, muscle	PKM	50.27	3	2	2
IBP4_HUMAN	Insulin like growth factor binding protein 4	IGFBP4	174.82	31	7	7
IBP7_HUMAN	Insulin like growth factor binding protein 7	IGFBP7	56.38	5	1	1
FETUA_HUMAN	Alpha 2-HS glycoprotein	AHSG	116.57	6	3	3
NEUA_HUMAN	Cytidine monophosphate N-acetylneuraminic acid synthetase	CMAS	27.45	1	1	1
PPIA_HUMAN	Peptidylprolyl isomerase A	PPIA	43.92	7	2	2

Continued

Uniprot accession	Protein name/Description	Gene symbol	-10lgP	Coverage (%)	#Peptides	#Unique
ALBU_HUMAN	Albumin	ALB	148	15	10	10
H0YKU7_HUMAN	Transient receptor potential cation channel subfamily M member 1	TRPM1	26.22	1	1	1
G3P_HUMAN	Glyceraldehyde-3-phosphate dehydrogenase	GAPDH	52.76	2	1	1
AL1A1_HUMAN	Aldehyde dehydrogenase 1 family member A1	ALDH1A1	78.21	5	3	3
ACTN4_HUMAN	Actinin alpha 4	ACTN4	27.13	1	1	1
ACTN1_HUMAN	Actinin alpha 1	ACTN1	27.13	1	1	1
ACTG_HUMAN	Actin gamma 1	ACTG1	162.11	13	4	4
Q1KLZ0_HUMAN	Actin beta	ACTB	162.11	13	4	4
AFAM_HUMAN	Afamin	AFM	25.38	1	1	1
BGH3_HUMAN	Transforming growth factor beta induced	TGFBI	54.81	3	2	2
AK1BA_HUMAN	Aldo-keto reductase family 1 member B10	AKR1B10	120.51	12	3	3
TKT_HUMAN	Transketolase	TKT	85.99	6	4	4
TYB10_HUMAN	Thymosin beta 10	TMSB10	33.86	18	1	1
B4DQ80_HUMAN	Tropomyosin 3	TPM3	43.44	8	2	2

Continued

Uniprot accession	Protein name/Description	Gene symbol	-10lgP	Coverage (%)	#Peptides	#Unique
1433T_HUMAN	Tyrosine 3-monooxygenase/tryptophan 5-monooxygenase activation protein theta	YWHAQ	35.85	4	1	1
1433Z_HUMAN	Tyrosine 3-monooxygenase/tryptophan 5-monooxygenase Activation protein zeta	YWHAZ	35.85	4	1	1
B5BU24_HUMAN	Tyrosine 3-monooxygenase/tryptophan 5-monooxygenase activation protein beta	YWHAB	35.85	4	1	1
Q0P5N8_HUMAN	Thymosin beta 4, X-linked	TMSB4X	86.89	39	2	2
TPM4_HUMAN	Tropomyosin 4	TPM4	43.44	6	2	2
Q6NS35_HUMAN	Eukaryotic translation elongation factor 1 alpha 1	EEF1A1	33.17	15	1	1
EPS15_HUMAN	Epidermal growth factor receptor pathway substrate 15	EPS15	27.45	1	1	1
EF1A3_HUMAN	Eukaryotic translation elongation factor 1 alpha 1 pseudogene 5	EEF1A1P5	33.17	2	1	1

List of detected proteins in A549 CD166-EpCAM- non-CSCs subpopulation derived from NSCLC, combined treatment sample.

Uniprot accession	Protein name/Description	Gene symbol	-10lgP	Coverage (%)	#Peptides	#Unique
C9JTX5_HUMAN	Actin beta	ACTB	66.41	15	1	1
GABR2_HUMAN	Gamma-aminobutyric acid type B receptor subunit 2	GABBR2	26.64	1	1	1
G3P_HUMAN	Glyceraldehyde-3-phosphate dehydrogenase	GAPDH	172.47	18	4	4
D0PNI1_HUMAN	Tyrosine 3-monooxygenase/tryptophan 5-monooxygenase activation protein zeta	YWHAZ	116.4	19	4	2
1433E_HUMAN	Tyrosine 3-monooxygenase/tryptophan 5-monooxygenase activation protein epsilon	YWHAE	75.43	12	3	1
E7ESG9_HUMAN	Transmembrane protease, serine 4	TMPRSS4	33.13	3	1	1
K7EJ44_HUMAN	Profilin 1	PFN1	56.32	11	1	1
Q7Z5F4_HUMAN	protease, serine 3	PRSS3	143.03	26	11	6
TRY6_HUMAN	Protease, serine 3 pseudogene 2	PRSS3P2	61.04	6	2	2
TKT_HUMAN	Transketolase	TKT	90.28	9	3	3
ALBU_HUMAN	Albumin	ALB	133.8	9	5	5
K1C9_HUMAN	keratin 9	KRT9	55.72	4	1	1
AK1BA_HUMAN	Aldo-keto reductase family 1 member B10	AKR1B10	113.05	9	2	2

Continued

Uniprot accession	Protein name/Description	Gene symbol	-10lgP	Coverage (%)	#Peptides	#Unique
CYTC_HUMAN	Cystatin C	CST3	53.88	6	1	1
ENOA_HUMAN	Enolase 1	ENO1	117.63	13	4	4
IBP4_HUMAN	Insulin like growth factor binding protein 4	IGFBP4	149.39	22	4	4
PRDX1_HUMAN	Peroxiredoxin 1	PRDX1	33.74	5	1	1
AL1A1_HUMAN	Aldehyde dehydrogenase 1 family member A1	ALDH1A1	132	11	5	5
Q5T3N1_HUMAN	Annexin A1	ANXA1	44.03	5	1	1
F8WB63_HUMAN	Actin, gamma 2, smooth muscle, enteric	ACTG2	66.41	11	1	1
I3L3I0_HUMAN	Actin gamma 1	ACTG1	74	6	1	1
Q5ZEZ4_HUMAN	Actinin alpha 1	ACTN1	51.37	14	1	1
ACTN4_HUMAN	Actinin alpha 4	ACTN4	51.37	1	1	1
F6QUT6_HUMAN	Actin, alpha 2, smooth muscle, aorta	ACTA2	66.41	8	1	1
A6NL76_HUMAN	Actin, alpha 1, skeletal muscle	ACTA1	66.41	4	1	1
